# The association between pulmonary tuberculosis recurrence and exposure to fine particulate matter and residential greenness: A population-based retrospective study

**DOI:** 10.1016/j.onehlt.2025.101035

**Published:** 2025-04-12

**Authors:** Yuanzhi Di, Ying Peng, Xiaogang Hao, Henan Xin, Tonglei Guo, Jiang Du, Xuefang Cao, Lingyu Shen, Juanjuan Huang, Yijun He, Boxuan Feng, Zihan Li, Jianguo Liang, Chunfu Fang, Ping Zhu, Yu Zhang, Fei Wang, Xiaomeng Wang, Bin Chen, Bingjun Xu, Lei Gao

**Affiliations:** a NHC Key Laboratory of Systems Biology of Pathogens, National Institute of Pathogen Biology, and Center for Tuberculosis Research, Chinese Academy of Medical Sciences and Peking Union Medical College, Beijing 102629, PR China; b Key Laboratory of Pathogen Infection Prevention and Control (Ministry of Education), National Institute of Pathogen Biology, Chinese Academy of Medical Sciences & Peking Union Medical College, Beijing 102629, PR China; c Center for Diseases Control and Prevention of Quzhou City, 324003, PR China; d Zhejiang Provincial Center for Diseases Control and Prevention, 310009, PR China; e Department of Neonatology, Shanghai Children’s Medical Center GuiZhou Hospital, Shanghai Jiao Tong University School of Medicine, No.166, Jinzhu East Road, Guanshanhu District, Guiyang 550081, PR China; f Department of Neonatology, Guizhou Provincial People’s Hospital, No.83, Zhongshan East Road, Nanming District, Guiyang 550002, PR China

**Keywords:** Air pollution, Greenness, PM_2.5_, Recurrence, Tuberculosis

## Abstract

**Background and objective:**

To assess the association of pulmonary tuberculosis (PTB) recurrence with fine particulate matter (PM_2.5_) and residential greenness using a population-based retrospective study design.

**Methods:**

All incident PTB patients, registered in Tuberculosis Information Management System (TBIMS) from 2015 to 2019 in Quzhou City, China, were included. The data on PM_2.5_ exposure was extracted from the China High Air Pollutants dataset and the level of greenness was estimated using the Normalized Difference Vegetation Index (NDVI) values around the patient's residence. The Cox proportional hazards models were used to quantify the risk of PTB recurrence.

**Results:**

6732 Eligible PTB incident patients were included in the study with a mean age of 56.86 years and a median follow-up time of 750 days. Recurrence was observed in 554 patients (8.2 %). Exposure to NDVI was observed to be negatively associated with PTB recurrence (HR: 0.86, 95 % CI: 0.75–0.98 per 0.1-unit increase). The strength of the association between higher PM_2.5_ and the risk of PTB recurrence was greater than that of lower PM_2.5_ concentrations in both low and high NDVI groups (HR:6.62 and 4.35, p-interaction <0.001).

**Conclusions:**

Our findings suggest that higher PM_2.5_ exposure might increase the risk of PTB recurrence, while residential greenness might have a protective effect. Like other chronic respiratory diseases, prevention and control of PTB will also benefit from comprehensive environmental management.

## Highlights

• Exposure of fine particulate matter increases pulmonary tuberculosis recurrence risk.
• Residential greenness negatively relates to pulmonary tuberculosis recurrence.
• Interaction might exist between residential greenness and fine particulate matter.

## 1 Introduction

Pulmonary tuberculosis (PTB) caused by the infection of *Mycobacterium tuberculosis* (MTB) is a major infectious disease and caused 1.30 million deaths worldwide in 2022 [1]. PTB recurrence, which stems primarily from relapse of the initial strain or reinfection with a new strain, is a significant contributor to the disease's burden especially in countries with severe epidemics [2,3]. Nearly half of PTB relapses occur within 1 to 3 years after prior treatment completion [4], with a 6.9 % recurrence rate observed in a 7-year follow-up study across 9 regions in China [5]. Effective control of recurrence is crucial not only for preventing PTB but also for mitigating the emergence of drug resistance. Numerous studies have identified that male, minority ethnicity, unemployment, migrant status, and positive bacteriological test results are associated with an increased risk of recurrent tuberculosis (TB) [6]. Air pollution ranks as the third largest cause of death and disability in China, following tobacco and high blood pressure [7]. A series of studies have observed that the impact of Fine Particulate Matter (PM_2.5_) on TB outcomes (incidence, recurrence, drug resistance and mortality) is stronger than that of other pollutants, which may be attributed to its physicochemical properties, especially its transition metal content and the mineral types of the particles. Studies have shown that the high levels of transition metal content, specifically iron, in PM_2.5_ were suggested to influence the proliferation of mycobacteria [8,9]. A nationwide study showed that higher residential greenness exposure levels can mitigate the impact of PM_2.5_ on TB incidence [10], underscoring the protective role of greenness against air pollution-related health risks. Research shows that individuals with multidrug-resistant tuberculosis (MDR-TB) could benefit from greenness, as it attenuates the associations between PM_2.5_ and mortality [11]. Furthermore, living close to green spaces is suggested to increase exposure to a variety of plant, animal, and microbial antigens, potentially bolstering resistance to infections and autoimmune responses [12]. However, at present, the scientific literature is devoid of studies exploring the association between environmental exposures and PTB recurrence. To bridge this gap, we launched a population-centric retrospective study to explore the link between PTB recurrence and exposure to air pollution and residential greenness, while also assessing the interactive effects of these two factors. The study's outcomes may provide new ideas for strengthening the comprehensive prevention and control of PTB and comorbidities.

## 2 Patients and methods

### 2.1 The study cohort

This retrospective study focused on patients with PTB identified between 2015 and 2019 in Quzhou, China. The prevalence of PTB in Quzhou aligns with the national average, yet it has the highest TB incidence rate within Zhejiang Province. All patients with PTB were identified and recorded in the Tuberculosis Information Management System (TBIMS), a national system covering all TB control institutions. It collects patient demographics, diagnostics, treatment management, outcomes, and especially provides us with details on initial or retreatment status [13]. The database captures an array of demographic and clinical details for each case, encompassing age, sex, occupation, current address, original residence, tuberculosis classification, date of PTB symptom onset, drug resistance result, diagnostic dates, and results from smear microscopy or culture. PTB cases identified by suspected clinical symptoms or radiographic abnormalities were referred for diagnosis according to the Chinese Health Industry Standard [14]. Briefly, the diagnosis of PTB was based on a combination of epidemiological history, clinical manifestations, radiographic findings, and laboratory tests, including sputum smear microscopy, mycobacterial culture, and molecular assays. Microbiologically confirmed cases were defined as those with positive results in any of the bacteriological tests. Clinically diagnosed cases were defined as those with negative bacteriological tests but typical clinical and radiographic features, and with positive response to anti-tuberculosis treatment. The criteria for including studies were as follows: Patients who were registered and completed treatment at a health facility in Quzhou between January 1, 2015, and December 31, 2019; aged 5 years or above; resident at the study location for no less than six months preceding their diagnosis; and the data on residential greenness and annual average PM_2.5_ concentration were available. Besides, we excluded migrant patients who moved out of Quzhou during the study period.

### 2.2 Outcome measures

In this study, PTB recurrence was defined as a new diagnosis of PTB between January 1, 2015, and December 31, 2019, confirmed either clinically or microbiologically, in patients who had previously been deemed cured or had completed treatment for an initial episode [15]. For patients who underwent initial treatment between 2015 and 2019, the follow-up period commenced from the date of completion of their initial treatment and extended until the date of recurrence. For those whose initial treatment was completed before 2015, the follow-up began on January 1, 2015, and continued until the date of recurrence. We only followed until their first recurrent PTB diagnosed. For the study participants without recurrence during the observation period, the follow-up period begins at the time of cure or treatment completion and ends at the follow-up endpoint or the time of death.

### 2.3 Estimates of exposure to PM_2.5_ and residential greenness

In assessing residential greenness, our methodology relied on the NDVI—a well-established metric derived from the Moderate-Resolution Imaging Spectroradiometer (MODIS) aboard NASA's Terra satellite. MODIS has a temporal resolution of 16 days and varying spatial resolution up to 500 m [16] and is employed in epidemiological research to determine the presence and density of outdoor vegetation [[17], [18], [19]]. The NDVI itself is a ratio, calculated by taking the difference in reflectance between the near-infrared band and the red visible spectrum and dividing it by their sum, yielding values that span from −1 to 1. Values between −1 and 0 typically denote aquatic environments, a zero value is indicative of bare soil, while readings from 0 to +1 for healthy green vegetation, with ascending values correlating with increased vegetative density [20]. We sourced the annual average PM_2.5_ concentration in Quzhou from 2015 through 2019 from the widely utilized China High Air Pollutants dataset, a resource prevalent in prior epidemiological research [21,22]. This integrated platform synthesizes satellite and ground-based observations, alongside demographic and geographical data, employing space-time extremely randomized trees models to achieve a fine spatial resolution of 1 km^2^ for PM_2.5_ estimations. The model's cross-validated coefficient of determination (R^2^) of 0.92 for yearly predicted estimate, with a root mean square error of 10.76 μg/m^3^ and a mean absolute error of 6.32 μg/m^3^ in daily PM_2.5_ predictions [23].In our study, we employed the Maximum Value Composite (MVC) technique to extract NDVI values, which was instrumental in reducing the impact of clouds and cloud shadows [24]. Furthermore, Evidence indicates an optimal radius for measuring accessible greenness in residential neighborhoods is approximately 0.25 miles [25]. The 500-m radius, being the closest approximation to 0.25 miles achievable with MODIS, is therefore utilized. Based on the latitude and longitude coordinates during the follow-up period of the patients (these addresses are sourced from the geocoded current addresses in the TBIMS, as shown in **eFigure1**), we extracted the annual average PM_2.5_ values and calculated the average value during the follow-up period. Additionally, we employed the MVC technique to process and assess greenness exposure. This technique significantly reduces the impact of clouds and cloud shadows [24] We also extracted NDVI values based on the latitude and longitude coordinates.

### 2.4 Individual and ground-based covariates

We adjusted for a range of individual covariates, including age at diagnosis (as 1-year strata), sex, occupation, drug resistance in tuberculosis (DR-TB), and county-level migrant population. Furthermore, we accounted for potential confounders related to traffic-related air pollution at the ground level, including distances to the nearest roads and the density of roads within a 500-m radius of the patients' residences, as well as the nighttime light (NTL) index. We sourced traffic data for national roads, highways, provincial roads, and residential streets from the OpenStreetMap road network, a resource previously validated in transportation research [26]. The NTL index served as a proxy for adjusting individual socioeconomic status and living conditions by reflecting urban development, human activities, and regional economic disparities [27]. Zhang Lixian et al. developed the Prolonged Artificial Nighttime Light Dataset of China using a convolutional Long Short-Term Memory network to enhance temporal NTL data continuity and reliability. The dataset, available from the National Tibetan Plateau Data Center, offers approximately 1 km spatial resolution with brightness values ranging from 0 to 63. Validation shows high quality, with a root mean square error of 0.73, a coefficient of determination of 0.95, and a nearly perfect pixel slope of 0.99 [28]. The yearly average NTL values were also calculated and linked to patients during the follow-up according to their current addresses.

### 2.5 Statistical analysis

Continuous variables with nonnormal distribution were expressed as median (interquartile ranges [IQRs]), and normally distributed variables were expressed as mean (standard deviations [SDs]). Categorical variables were expressed as numbers (percentages) for baseline characteristics. Differences in the frequency of the PTB recurrence according to these variables were assessed by Pearson's chi-square test. The association of PTB recurrence with exposure to PM_2.5_ and residential greenness was estimated with Hazard ratios (HRs) and 95 % confidence intervals (CIs) using Cox proportional hazards models. Estimates of association were adjusted for age at diagnosis and sex, and, in addition, for other covariates that were previously reported to be associated with PTB including occupation, county-level migrant population, drug resistance, NTL, distances to nearest roads, and road density [29]. To address the competing risk of death, we utilized the Fine and Gray subdistribution hazards regression model for analysis, with covariates consistent with those included in the preceding multivariable Cox models. We also stratified PM_2.5_ and NDVI levels into quartiles based on their interquartile ranges and performed trend tests by treating these categories as continuous variables in Cox proportional hazards models. Subgroup analyses were conducted, stratified by age, sex, occupation, NTL group, and districts, in the previous fully adjusted model. Patients were categorized into low versus high urbanization areas using the median NTL value as a cut-off. Multiplicative interaction evidence between NDVI and PM_2.5_ was assessed using the likelihood ratio test by comparing Cox proportional hazards models with and without interaction terms. To assess the dose-response relationships between the risk of PTB recurrence with the exposure levels of PM_2.5_ and greenness, we employed the Cox proportional hazards models with restricted cubic splines. Knots were set at the 10th, 50th, and 90th percentiles of PM_2.5_ and greenness levels. Nonlinearity was assessed using Wald statistics. Covariates in these dose-response models mirrored those used in the preceding multivariable Cox models. To exclude the possibility of misdiagnosis in pathogen-negative clinically diagnosed cases, sensitivity analyses were carried out by focusing on patients with diagnosed with microbiological evidence. Because treatment failure and early relapse are actually a continuum, we conducted a sensitivity analysis focusing on patients with a microbiological diagnosis and excluding cases with follow-up of less than 90 days [30]. Statistical evaluations were conducted with ArcGIS 10.2 (ESRI, Redlands, CA, USA) and R version 4.3.3 (R Project for Statistical Computing) with the analysis packages survival, version 3.5–8; dplyr, version 1.1.4; ggplot2, version 3.5.0; raster, version 3.6–26; lubricate, version 1.9.3; forestplot, version 3.1.3. All statistical tests were 2-sided, and a *p*-value < .05 was considered statistically significant.

## 3 Result

### 3.1 Study population

A total of 8490 patients were registered between 2015 and 2019 in Quzhou. After excluding 472 migrants (5.56 %), 232 residents (2.73 %) with missing residential records or data on air pollution or NDVI, 1052 patients (12.39 %) who did not complete treatment before 2019, as well as 2 individuals (0.02 %) under the age of five, the final sample comprised 6732 participants with a median age of 61 years (IQR: 44–71), as shown in **eFigure3**. Among these participants, there were 554 PTB recurrences during the follow-up period, with a median follow-up time of 750 days per patient. A total of 548 patients with a history of PTB diagnosis, who were asymptomatic at baseline, were confirmed to have PTB recurrence during the follow-up period. Characteristics of the participants are presented in Table 1 and **eFigure1**. Over half of the patients (53.96 %, 3565/6732) were aged≥60 years, with a male predominance (70.42 %, 4741/6732). The majority (71.70 %, 4827/6732) were engaged in agriculture. DR-TB was identified in 1.77 % (119/6732) of cases. The number of microbiologically confirmed cases is 2753(40.89 %). The distribution of PTB recurrence differed significantly across various factors including sex, occupation, DR-TB status, different districts, and NTL groups. The geographical analysis revealed an uneven distribution of TB cases across districts. **eFigure2** shows the mean distribution of NDVI, PM_2.5_, and NTL among individuals in different regions.

**Table 1:** Characteristics of recurrent pulmonary tuberculosis patients occurred from 2015 to 2019 in Quzhou, China.

Characteristics	No. (%) /Median (IQR)	Recurrent PTB No. (%)	*p* for difference
Total	6732	554(8.23)	
Age at diagnosis date (years)			<0.001
0–59.9	3167(47.04)	175(5.53)	
≥60	3565(52.96)	379(10.63)	
Sex			0.001
Female	1991(29.58)	130(6.53)	
male	4741(70.42)	424(8.94)	
Occupation			0.005
Farming	4827(71.70)	426(8.83)	
Others	1905(28.30)	128(6.72)	
Drug-resistant status			<0.001
Yes	119(1.77)	31(26.05)	
No	6613(98.23)	523(7.91)	
Distance to the nearest roads⁎			0.22
Low	0.02(0.01–0.02)	276(8.03)	
High	0.07(0.04–0.11)	278(8.43)	
Residential road density‡			0.07
Low	2.84(2.05–3.53)	299(8.88)	
High	6.24(5.16–8.48)	255(7.58)	
Residential NTL§			0.88
Low	5.8(1.93–6.85)	280(8.3)	
High	23.52(11–37.62)	274(8.15)	
Residential PM_2.5_⁂			<0.001
Low	28.8(27.5–29.9)	50(1.48)	
High	33.8(32.5–35.85)	504(15)	
Residential NDVI†			0.31
Low	0.65(0.56–0.70)	289(8.59)	
High	0.79(0.76–0.83)	265(7.87)	
Location by District			<0.001
Changshan	1252(18.60)	91(7.27)	
Jiangshan	1572(23.35)	89(5.66)	
Longyou	883(13.12)	109(12.34)	
Kaihua	947(14.07)	74(7.81)	
Kecheng	1031(15.31)	92(8.92)	
Qujiang	1047(15.55)	99(9.46)	

Abbreviations: CI, confidence interval; IQR, interquartile range; NDVI, normalized difference vegetation index; NTL, nighttime light; PM_2.5_, fine particulate matter; PTB, pulmonary tuberculosis.

⁎ Distances to the nearest road use a median value of 0.03 km as the low-high cutoff.

§ Yearly average nighttime light index was used as a proxy for socioeconomic level and urbanization. Residential NTL uses a median value of 8.09 as the low-high cutoff.

⁂ Residential PM_2.5_ represents the air pollution level with a median value of 31.20 μg/m^3^ as the low-high cutoff.

† Residential NDVI represents the greenness level with a median value of 0.73 as the low-high cutoff.

‡ Measured as the length of roads divided by the total areas of the 500 m buffer around residential addresses. Residential road density uses a median value of 4.23 km/km2 as the low-high cutoff.

### 3.2 PM_2.5_ Exposures and PTB Recurrence

**Fig. 1:**
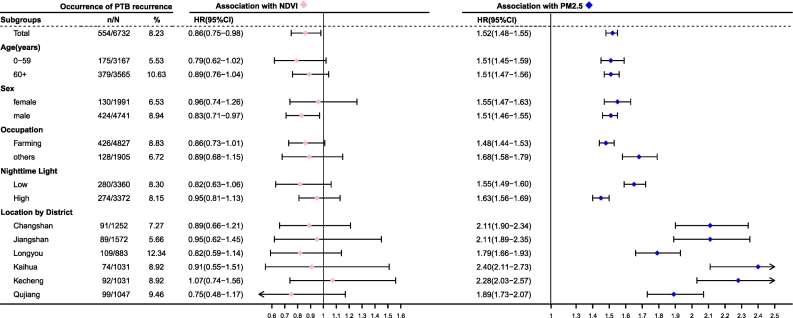
Subgroup analysis on the associations of pulmonary tuberculosis recurrence with residential greenness and fine particulate matter. Note: This figure presents the Cox proportional hazards model analysis results examining the association between residential greenness/fine particulate matter (PM_2.5_) and pulmonary tuberculosis recurrence risk. Age at the diagnosis date, sex, occupation, county-level migrant population, drug resistance, residential nighttime light (NTL), distance to the nearest roads, and residential road density were adjusted for in the Cox model. For statistical analysis, we utilized 0.1-unit increments for Normalized Difference Vegetation Index (NDVI). We use a forest plot to show the result. Forest plot illustrating the hazard ratios (HRs) and 95 % confidence intervals (CIs) for various subgroups. Subgroups are analyzed by age groups, sex, occupation, residential nighttime light groups, and location by district. Residential nighttime light uses the median value of 8.09 as the low-high cutoff.

**Fig. 2:**
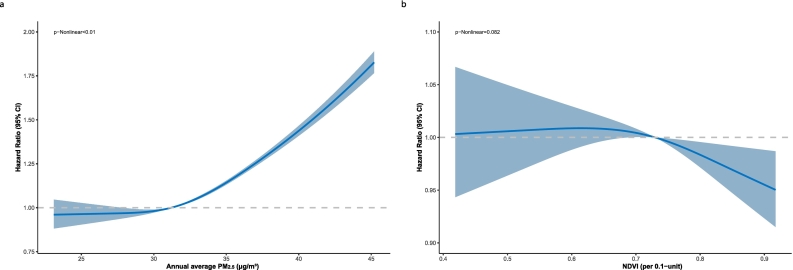
Dose-response association between environment exposure and the risk of pulmonary tuberculosis recurrence. Note: (a)The multivariate-adjusted HR are shown for the associations between annual average PM_2.5_ exposure and the risk of pulmonary tuberculosis (PTB) recurrence. (b) The multivariate-adjusted HR are shown for the associations between greenness exposure and the risk of PTB recurrence. The Cox model was adjusted for Age at the diagnosis date, sex, occupation, county-level migrant population, drug resistance, residential nighttime light, distance to the nearest roads, and residential road density. Dark curves and pale blue areas show predicted HRs and 95 % CIs, respectively. Abbreviation: CI, confidence interval; HR, hazard ratio; NDVI, normalized difference vegetation index; PM_2.5_, fine particulate matter. (For interpretation of the references to colour in this figure legend, the reader is referred to the web version of this article.)

Table 2 shows HRs with 95 %CIs for PTB recurrence in two adjusted models. Compared with group Q1 (reference group) with the lowest quintile of PM_2.5_, Quartile 3 had a proportion of patients with recurrence of 3.87 % (65/1679), with HRs of 2.07 (95 % CI: 1.20–3.59) and 2.44 (95 % CI: 1.41–4.24). Quartile 4 showed a higher proportion of patients with recurrence of 26.10 % (439/1682), with HRs of 10.52 (95 % CI: 6.36–17.39) and 15.48 (95 % CI: 9.28–25.82). Furthermore, a clear dose-response relationship between PM_2.5_ and the risk of PTB recurrence (*P* < .001) across two adjusted models. And a nonlinear dose-response relationship with an increasing trend between PM_2.5_ exposure levels and the risk of PTB recurrence (*P* < .01), while a linear dose-response relationship exists between greenness exposure levels and the risk of PTB recurrence (*P* = .082) in Fig. 2. The Fine and Gray model found that higher NDVI reduced recurrent PTB risk (SHR: 0.863, 95 % CI: 0.754–0.987), while increased PM_2.5_ raised risk (SHR: 1.512, 95 % CI: 1.475–1.550), matching Cox regression findings. The strength of the associations was more pronounced among individuals with lower NTL exposure and non-farmers as illustrated in Fig. 1.

**Table 2:** The association of pulmonary tuberculosis recurrence with exposure to residential greenness and fine particulate matter.

Exposures	n/N	%	Adjusted HR (95 % CI)⁎	Adjusted HR (95 % CI)‡
Residential NDVI				
Quartile1 (0.39, 0.65)	132/1683	7.83	Ref.	Ref.
Quartile2 (0.65, 0.73)	157/1680	9.33	1.10 (0.87–1.38)	1.06 (0.79–1.43)
Quartile3 (0.73, 0.80)	145/1683	8.60	0.93 (0.73–1.18)	0.89 (0.64–1.25)
Quartile4 (0.80, 0.94)	120/1683	7.12	0.81 (0.64–1.04)	0.74 (0.52–1.06)
*p* for Trend			0.05	0.02
Residential PM_2.5_				
Quartile1 (22.00, 28.80)	16/1720	0.93	Ref.	Ref.
Quartile2 (28.80, 31.20)	34/1651	2.06	1.59 (0.88–2.88)	1.78 (0.98–3.23)
Quartile3 (31.20, 33.77)	65/1679	3.87	2.07 (1.20–3.59)	2.44 (1.41–4.24)
**Quartile4 (33.77, 47.10)**	**439/1682**	**26.10**	**10.52 (6.36–17.39)**	**15.48 (9.28–25.82)**
*p* for Trend			<0.001	<0.001

Abbreviations: CI, confidence interval; HR, hazard ratio; NDVI, normalized difference vegetation index; PM_2.5_, fine particulate matter.

⁎ Age at the diagnosis date and sex were adjusted for in the Cox model.

‡ Age at the diagnosis date, sex, occupation, county-level migrant population, drug resistance, residential nighttime light, distance to the nearest roads, and residential road density were adjusted for in the Cox model.

### 3.3 Greenness Exposures and PTB Recurrence

While the association between NDVI and PTB recurrence was not statistically significant in Table 2, a trend emerged when stratifying the data by NDVI quartiles, suggesting that increased greenness may be linked to a lower risk of PTB recurrence. Similar associations between PM_2.5_ or NDVI and the risk of PTB recurrence were observed in the sensitivity analysis (**eTable1** and **eTable2** in the Supplement) and in the multivariable Cox regression that included other environmental factors (**eTable4** in the Supplement). As shown in Fig. 1, each 0.1-unit increase in greenness was associated with a HR of 0.86 (95 % CI: 0.75–0.98) in multivariate analyses. The associations were more pronounced among individuals engaged in farming and those with lower exposure to NTL.

### 3.4 Interaction between exposure to PM_2.5_ and residential greenness

As shown in Table 3, the interaction between NDVI and PM_2.5_ levels on the recurrence of PTB was systematically assessed, revealing significant associations across different exposure categories (*p* < .001). Within the same NDVI category, the proportion of individuals exposed to elevated levels of PM_2.5_ was markedly higher compared to those with lower PM_2.5_ concentrations. In both high and low NDVI regions, exposure to elevated PM_2.5_ was associated with increased risk. Specifically, in low NDVI areas, the fully-adjusted HR was 6.62 (95 % CI: 4.12–10.64), while in high NDVI areas, the HR was 4.35 (95 % CI: 2.84–6.66), relative to the reference group with lower PM_2.5_ levels.

**Table 3:** Interaction between residential greenness and fine particulate matter on modifying the pulmonary tuberculosis recurrence risk.

Residential NDVI⁎	Residential PM_2.5_‡	Recurrent PTB		Adjusted HR (95 % CI)§	Adjusted HR (95 % CI)⁂
		n/N	%		
Low	Low	20/1694	1.18	Ref.	Ref.
	High	269/1672	16.09	5.83 (3.68–9.26)	6.62 (4.12–10.64)
High	Low	24/1685	1.42	Ref.	Ref.
	**High**	**241/1681**	**14.34**	**4.42 (2.87–6.80)**	**4.35 (2.84–6.66)**
*p* for interaction				<0.001	<0.001

Abbreviations: CI, confidence interval; HR, hazard ratio; NDVI, normalized difference vegetation index; PM_2.5_, fine particulate matter; PTB, pulmonary tuberculosis.

⁎ Residential NDVI represents the greenness level with a median value as the low-high cutoff.

‡ Residential PM_2.5_ represents the air pollution level with a median value as the low-high cutoff.

§ Age at the diagnosis date and sex were adjusted for in the Cox model.

⁂ Age at the diagnosis date, sex, occupation, county-level migrant population, drug resistance, residential nighttime light, distance to the nearest roads, and residential road density were adjusted for in the Cox model.

## 4 Discussion

To our knowledge, this is the first study in China to explore the association of PTB recurrence risk with residential greenness and air pollution exposure. Our findings suggest that PM_2.5_ exposure is significantly associated with an increased risk of PTB recurrence, higher levels of residential greenness are linked to a decreased risk of PTB recurrence. Furthermore, our research highlights a statistically significant interaction between residential greenness and air pollution in modulating the risk of PTB recurrence.

The significantly positive association between PM_2.5_ exposure and PTB recurrence observed in our study is consistent with previous studies [31,32] and epidemiological findings. A multi-city modeling study in Shandong Province found that each 1 μg/m^3^ increase in PM_2.5_ was associated with a 0.55 % rise in recurrent TB cases [33]. Meanwhile, a cohort study in Zhengzhou revealed that each 10 μg/m^3^ increase in PM_2.5_ significantly elevated the risk of TB retreatment, with a HR of 1.97 [9]. High PM_2.5_ exposure in the fourth quartile significantly raises the risk of tuberculosis recurrence, potentially due to a non-linear relationship (Fig. 2) where health risks intensify beyond a certain PM_2.5_ threshold. The cumulative effect of high PM_2.5_ levels could also explain this [34,35]. Additionally, prior anti-tuberculosis treatment significantly contributes to the occurrence of drug resistance with higher incidence observed in recurrent PTB patients compared to newly treated individuals. Our study found that elevated PM_2.5_ levels are associated with an increased risk of developing DR-TB, as shown in **eTable3**. The potential biological mechanisms through which exposure to environmental PM_2.5_ adversely affects PTB are as follows: Air pollution exerts a deleterious impact on pulmonary function and the immune system by diminishing macrophage activity, instigating oxidative stress and inflammation, and amplifying reactivity [36,37]. The oxidative stress instigated by the PM has the potential to impair the airway epithelium and attenuate immune defenses against MTB [38]. Furthermore, these mechanisms may be particularly pronounced in patients with a history of TB. Based on previous studies, the impact of particulate matter on MTB-induced peripheral blood mononuclear cell (PBMC) responses was examined against a background of prior MTB infection-induced immune memory. The study found a higher frequency of MTB antigen-specific IFN-γ-producing PBMCs in subjects with prior MTB infection [39]. Additionally, it was shown that PM_2.5_ can cause pulmonary inflammation and fibrosis [40]. An epidemiological study revealed a complex relationship between PM2.5 levels and the standardized incidence rates (SIR) of TB. While PM2.5 had no significant impact on new TB SIR, there were significant positive correlations between PM2.5 levels and the SIR of recurrent TB [41]. Our findings indicate that individuals in high NTL areas are more impacted by air pollutants due to increased industrial activities and vehicular emissions, making these regions hotspots for high PM_2.5_ concentrations, with secondary industry share in the gross domestic product (GDP) being the primary determinant of annual average PM_2.5_ levels in China [42]. We effectively illustrate how environmental exposures differ geographically, providing a clearer understanding of the spatial disparities in environmental quality and their potential implications for public health.

Our findings suggest that each 0.1-unit increase in NDVI is associated with a 24 % lower risk of PTB recurrence, indicating a protective effect of greenness. The study participants who reside in areas with more greenness had lower socioeconomic status owing to China's urbanization and economic development [43]. This might skew our effect estimates towards null, aligning with two cohort studies on residential greenness and mortality in China [19,44]. This also elucidates our observation that the protective influence of green spaces was more pronounced among those experiencing lower levels of NTL. This finding aligns with a recent study conducted in China, demonstrating a robust inverse correlation between the NDVI and NTL [29]. In our study, we retrospectively included all PTB patients from 2015 to 2019. The proportion of males was relatively high, which is consistent with the overall local incidence [1,6]. To control for the influence of sex on the outcomes, we conducted a stratified analysis and found the impact of environmental factors on PTB recurrence was not significantly different by sex.

The harmful effects of PM_2.5_ was found to be vary with NDVI levels suggesting that low vegetation cover may worsen air pollution's health impacts. Moreover, the observation that people engaged in farming benefit more from residential greenness and are less adversely affected by PM_2.5_ compared to non-farmers further supports this point. A cross-sectional survey found that higher NDVI was associated with a 25 % lower risk of spirometric restriction, underscoring the protective effect of NDVI on lung health in low-to-moderate pollution areas [45]. A study in Anhui, China, demonstrated that individuals with higher median exposure to green landscapes had a 13.5 % lower risk of PM_2.5_-related mortality per 10 μg/m^3^ [46]. These findings are consistent with our research. Possible mechanisms are as follows: Areas with high densities of trees and vegetation can capture PM_2.5_ on their leaves, thereby reducing air pollution [47]. Further atmospheric chemistry studies have shown that vegetation not only intercepts airborne particles but also absorbs gaseous pollutants through the stomata on leaf surfaces [48]. Other studies present mixed results and lack consensus on whether greenness truly mediates the pathways of air pollution-related exposure [49,50]. The interaction between PM_2.5_ and greenness exposure further indicate that environmental factors influence infectious diseases outcomes in complex and interrelated ways. Individual interventions, such as avoiding outdoor activities during peak traffic hours or near main roads, reducing exercise intensity when air pollution is high [51], wearing protective masks to reduce PM_2.5_ inhalation [52], choosing residences in communities with high-quality greenery [53], and utilizing public green spaces [53], may mitigate environmental risks.

The reported incidence of PTB in Zhejiang Province is at an intermediate level nationwide. However, Quzhou faces a relatively high risk of PTB, which may be linked to local environmental factors [54]. Our large-sample study further confirms the association between environmental conditions and the recurrence risk of PTB. As we all know, even with successful treatment, risk factors persist. Therefore, reducing PM_2.5_ levels and increasing greenness may yield significant benefits by decreasing PTB notifications, particularly given the widespread exposure to such pollutants in Quzhou.

Our study is not without its limitations. First, despite the exclusion of all migrant patients from our analyses, the issue of neighborhood self-selection bias may be influenced by socioeconomic status, with more greenness often associated with lower socioeconomic status in China. We also did not account for the impact of residential changes due to a lack of detailed postal code data. Second, while the NDVI data offers valuable information on the quantity and presence of vegetation, it unfortunately lacks insights into the specific type or quality of vegetation. This limitation precludes the differentiation between urban green spaces and rural agricultural areas. Third, factors such as body mass index, education, smoking, other personal health behaviors, outdoor activity time, and indoor use of solid fuels were not included, despite adjusting for multiple covariates. Both individual-level behaviors and community-level socioeconomic factors were not fully considered. Future studies with more comprehensive data should explore these interactions to ensure a more comprehensive and accurate interpretation of results. Fourth, the time-to-event calculation may be biased because individuals completing treatment before 2015 have shorter follow-up periods, potentially underestimating recurrence time and affecting HR estimates. Fifth, for patients with a history of prior TB before 2015, our assessment only considered exposure from January 1, 2015, onward. This method fails to cover the entire period from the end of their previous treatment to TB recurrence. Hence, the exposure calculation in this study mainly reflects recent exposure, not the accumulated exposure over time.

## 5 Conclusions

Our study found that PM_2.5_ exposure significantly increases the risk of PTB recurrence, whereas increased greenness is associated with a reduced risk. More importantly, our study reveals a significant interaction between residential greenness and PM_2.5_ concentration. Specifically, increased greenness appears to mitigate the detrimental effects of PM_2.5_ on PTB recurrence, indicating that combined efforts in enhancing residential greenness and reducing air pollution are vital for preventing PTB recurrence. For high-risk groups such as individuals with prior PTB, individual intervention against environmental risk factors may also become one of the effective means of comprehensive prevention and control strategies.

## Data sharing statement

Survey data are available from the corresponding authors upon reasonable request.

## CRediT authorship contribution statement

**Yuanzhi Di:** Writing – original draft, Validation, Software, Methodology, Investigation, Conceptualization. **Ying Peng:** Supervision, Resources. **Xiaogang Hao:** Supervision, Resources. **Henan Xin:** Resources, Methodology. **Tonglei Guo:** Resources, Methodology. **Jiang Du:** Resources, Methodology. **Xuefang Cao:** Resources, Methodology. **Lingyu Shen:** Resources, Methodology. **Juanjuan Huang:** Resources. **Yijun He:** Resources. **Boxuan Feng:** Resources. **Zihan Li:** Resources. **Jianguo Liang:** Resources. **Chunfu Fang:** Resources. **Ping Zhu:** Software. **Yu Zhang:** Resources. **Fei Wang:** Resources. **Xiaomeng Wang:** Supervision, Resources. **Bin Chen:** Validation, Supervision, Software, Resources. **Bingjun Xu:** Investigation, Supervision, Resources. **Lei Gao:** Writing – review & editing, Supervision, Methodology, Investigation, Funding acquisition.

## Ethics approval

The ethics committees of the Institute of Pathogen Biology and the Chinese Academy of Medical Sciences approved the current study (IPB-2024-12).

## Author agreement statement

We declare that this manuscript is original, has not been published before and is not currently being considered for publication elsewhere.

We confirm that the manuscript has been read and approved by all named authors and that there are no other persons who satisfied the criteria for authorship but are not listed. We further confirm that the order of authors listed in the manuscript has been approved by all of us.

We understand that the Corresponding Author is the sole contact for the Editorial process.He is responsible for communicating with the other authors about progress, submission of revisions and final approval of proofs.

## Funding/support

This work was supported by the CAMS Innovation Fund for Medical Sciences (CIFMS) [2021-I2M-1-037] and the Non-profit Central Research Institute Fund of Chinese Academy of Medical Sciences [2023-PT310-04].

## Role of the funder/sponsor

The funders had no role in the design and conduct of the study; collection, management, analysis, and interpretation of the data; preparation, review, or approval of the manuscript; and decision to submit the manuscript for publication.

## Declaration of competing interest

The authors declare that they have no known competing financial interests or personal relationships that could have appeared to influence the work reported in this paper.

## Data Availability

Data will be made available on request.

## Appendix A Supplementary data

Supplementary material Image 1
