# Supplementary material for: The association between pulmonary tuberculosis recurrence and exposure to fine particulate matter and residential greenness: A population-based retrospective study
Source: One Health. 2025 Apr 12;20:101035. doi: 10.1016/j.onehlt.2025.101035 (PMC12047573; doi:10.1016/j.onehlt.2025.101035)
Supplement: Supplementary file 1 — Supplementary material [file mmc1.docx]

**The association between Pulmonary Tuberculosis Recurrence and the exposure to Fine Particulate Matter and Residential Greenness: A Population-Based Retrospective Study**

**S-Methods**

The association of drug-resistant tuberculosis (TB) occurrence with exposure to fine particulate matter (PM_2.5_) and residential greenness was estimated with odds ratios (ORs) and 95% confidence intervals (CIs) using logistic regression models. Estimates of association were adjusted for age at diagnosis and sex, and, in addition, for other covariates that were previously reported to be associated with TB including occupation, county-level migrant population, nighttime light (NTL), distances to nearest roads, and road density[1]. We also stratified PM_2.5_ and Normalized Difference Vegetation Index (NDVI) levels into quartiles based on their interquartile ranges and performed trend tests by treating these categories as continuous variables in logistic regression models. Additionally, we control for other environmental pollutants and meteorological factors in the final multivariable models. The air pollutant datasets employ the extensively utilized and comprehensive [China High Air Pollutants](https://zenodo.org/communities/chap/) (CHAP) datasets, which provide high-resolution, long-term, full-coverage data on ground-level atmospheric pollutants across China. The CHAP datasets encompass near-surface air pollutants including suspended particulate matter and gaseous pollutants. For PM_2.5_ and PM_10_, the spatial resolution is 1 km, with coefficients of determination (R²) of 0.92 and 0.89, and root mean square errors (RMSE) of 10.76 µg/m³ and 21.12 µg/m³, respectively[2, 3]. Gaseous pollutants such as Sulfur Dioxide (SO₂), and Nitrogen Dioxide (NO₂) had a spatial resolution of 10 km, their R² values are 0.84, and 0.84, with RMSEs of 0.29 µg/m³, 7.99 µg/m³, and 4.89 µg/m³, respectively[4, 5]. The Ozone (O_3_)[6] data features a spatial resolution of 1 km, boasting a ten-fold cross-validation R² of 0.89 and an RMSE of 15.77 µg/m³. The temperature data comes from the monthly average temperature data of China, with a spatial resolution of approximately 1 km[7]. The relative humidity data comes from A 1 km high-resolution atmospheric moisture index collection over China (HiMIC-Monthly), with a temporal resolution of 1 month and a spatial resolution of 1 km[8]. The association of PTB recurrence with exposure to PM_2.5_ and residential greenness was estimated using Cox proportional hazards models, with hazard ratios (HRs) and 95% CIs as the measures of association. The models were adjusted for age at diagnosis and sex, as well as for other covariates previously reported to be associated with PTB, including occupation, county-level migrant population, drug resistance, NTL, distances to the nearest roads, road density, O_3_, SO_2_, temperature, and relative humidity. We extract the latitude and longitude coordinates for each day based on the address, and calculate the average daily pollutant levels and monthly temperature, and relative humidity levels over the follow-up period according to the follow-up time. Due to multicollinearity issues, which increased the number of iterations and prevented stable convergence of the Cox regression models, certain variables were excluded from the final model to ensure feasibility and stability. Specifically, PM_10_ was not included due to its strong correlation with PM_2.5_ (r = 0.846), and NO_2_ was also excluded because of its significant correlation with PM_2.5_ (r = 0.694) and its moderate negative correlation with NDVI (r = -0.665). These exclusions were necessary to maintain the robustness and reliability of the model estimates.**Supplementary materials. (eFigure 1-3; eTable 1-4)**

**eFigure1.** The distribution characteristics of pulmonary tuberculosis, and residential greenness in Quzhou, Zhejiang, China

**eFigure2.** Living environment characteristics of pulmonary tuberculosis patients in different districts in Quzhou, China

**eFigure 3.** Flowchart of patient selection and data collection process

**eTable1.** The association of microbially confirmed PTB with exposure to residential greenness and PM_2.5_

**eTable2.** Pulmonary tuberculosis and exposure to residential greenness and PM_2.5_, excluding follow-ups less than 90 days

**eTable3.** The association of drug-resistant tuberculosis occurrence with exposure to residential greenness and PM_2.5_

**eTable4.** The association of pulmonary tuberculosis recurrence with exposure to residential greenness and PM_2.5_

**eFigure 1. The distribution characteristics of pulmonary tuberculosis, and residential greenness in Quzhou, Zhejiang, China**

Note: The study site, Quzhou City, has the highest TB incidence rate in Zhejiang Province. The map displays the location of PTB cases recorded from 2015 to 2019 alongside residential greenness exposure, defined as the 16-day average Normalized Difference Vegetation Index (NDVI) within a 500-meter buffer around residential addresses during the follow-up period.


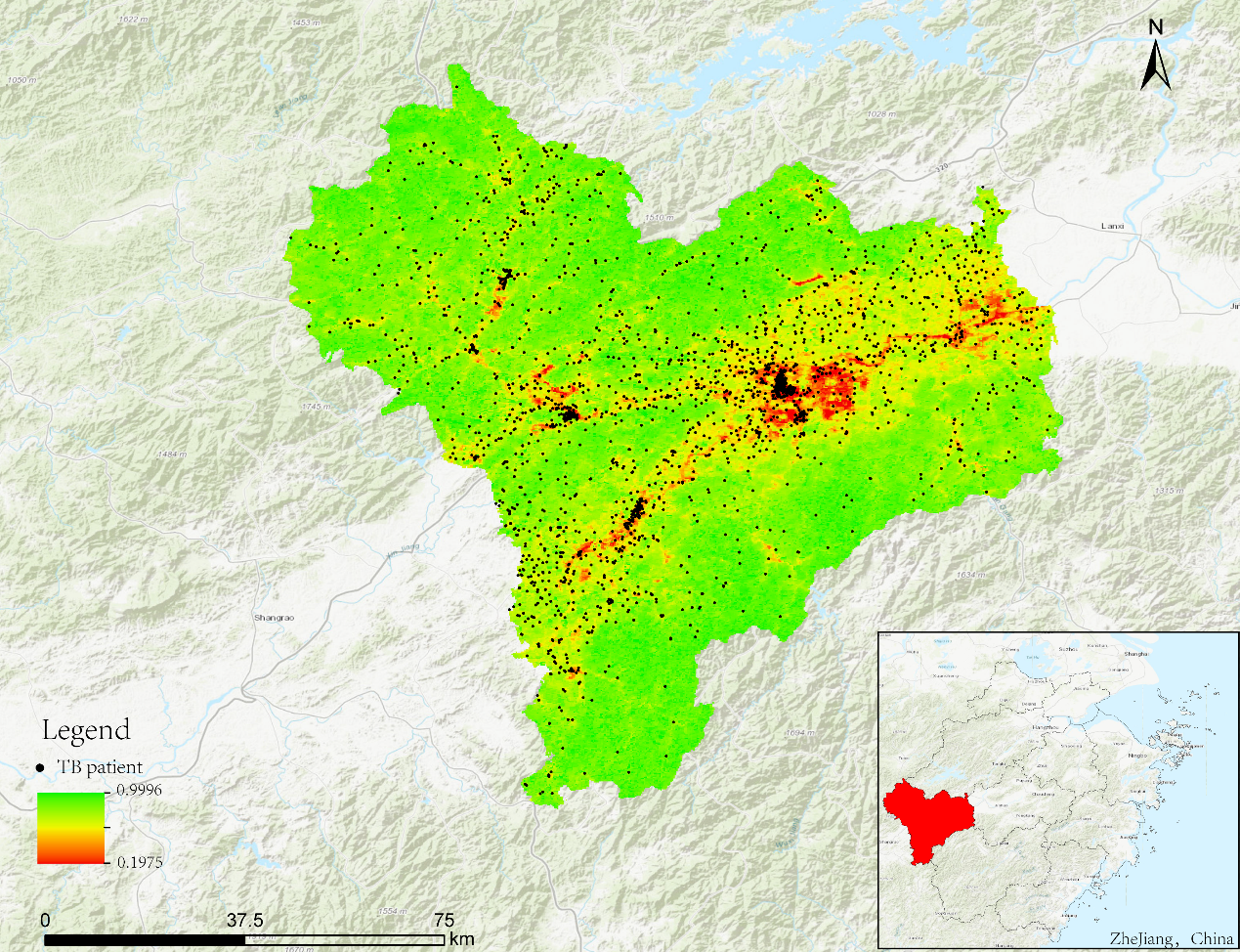


**eFigure 2. Living environment characteristics of pulmonary tuberculosis patients in different districts in Quzhou, China**

Note: The Distributions of different environmental factors in various districts of Quzhou City were shown: (a) Residential PM_2.5_ concentration; (b) Residential Normalized Difference Vegetation Index (NDVI); (c) Residential Nighttime Light Index (NTL). Each violin plot represents the distribution of the respective factor across six districts: Changshan, Jiangshan, Kaihua, Kecheng, Longyou, and Qujiang. The box plots within the violins indicate the interquartile ranges and median values.


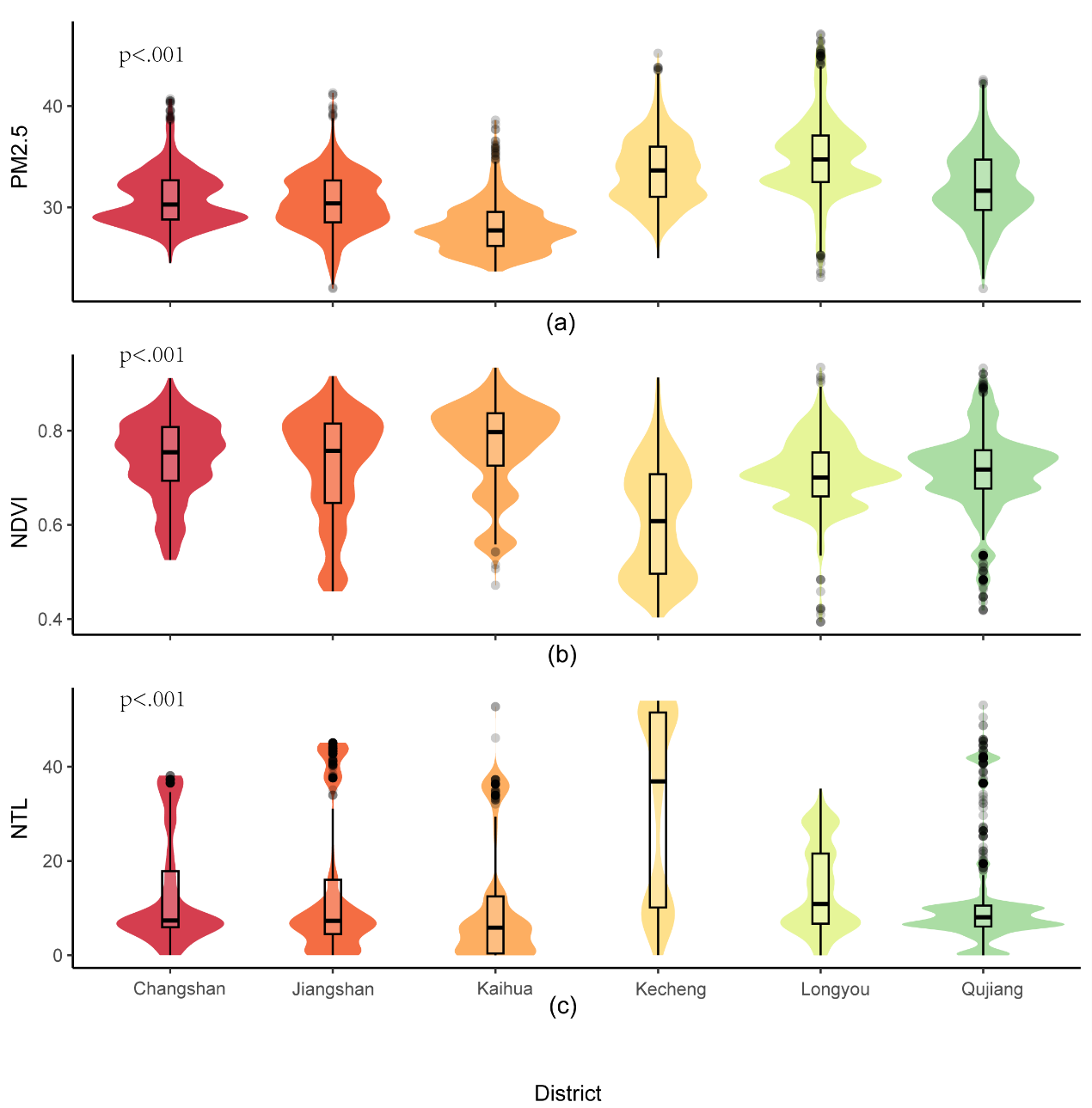


**eFigure 3. Flowchart of patient selection and data collection process**The flowchart illustrates the stepwise inclusion and exclusion of PTB (pulmonary tuberculosis) patients in the study conducted at the Quzhou Center for Disease Control and Prevention from 2015 to 2019.

**
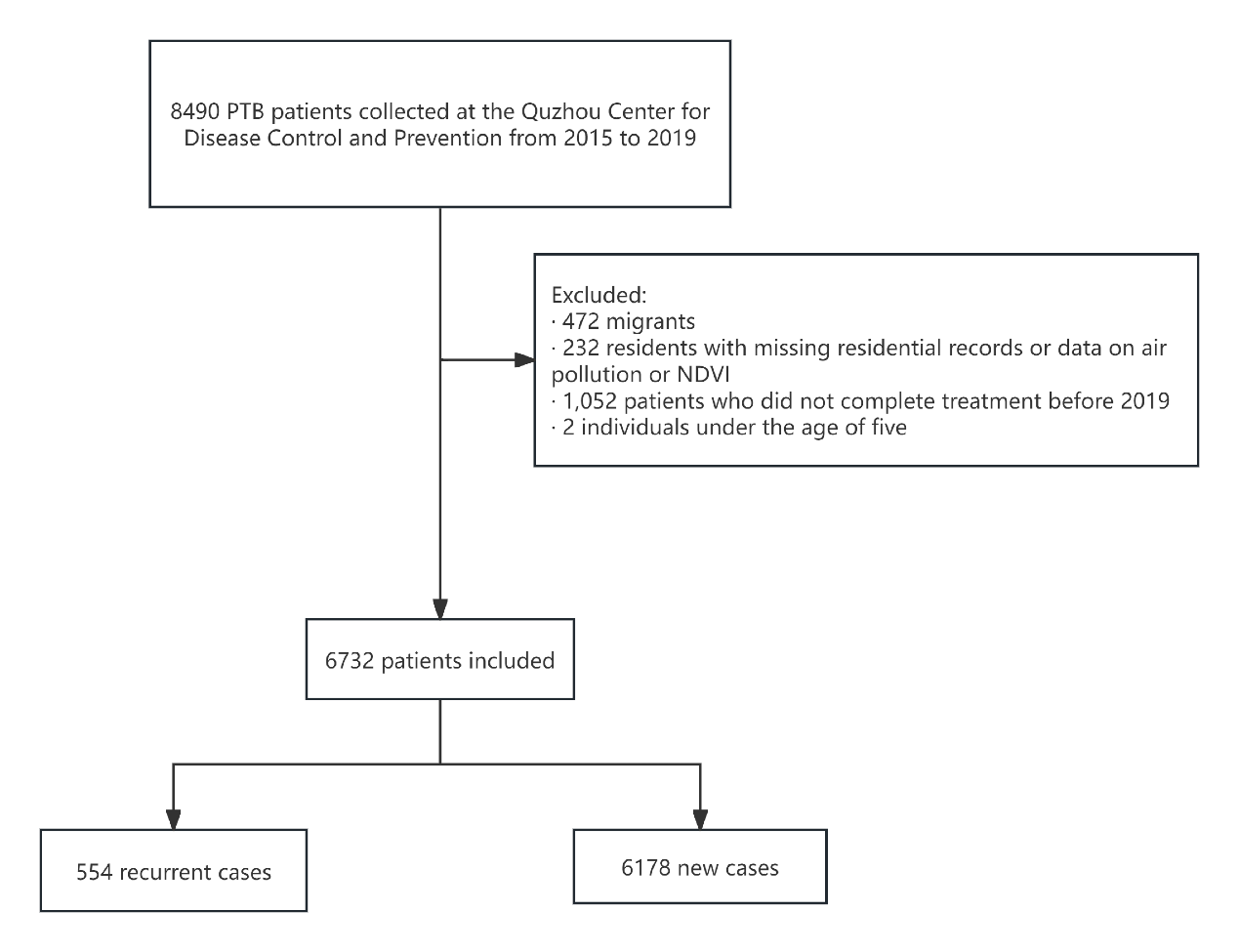
**

**eTable1. The association of microbially confirmed pulmonary tuberculosis with exposure to residential greenness and PM_2.5_**

| Exposures | n/N | % | Adjusted HR (95% CI) ^*^ | Adjusted HR (95% CI) ^‡^ |
| --- | --- | --- | --- | --- |
|  |  |  |  |  |
| Residential NDVI |  |  |  |  |
| Quartile1 (0.39, 0.65) | 77/694 | 11.10 | Ref. | Ref. |
| Quartile2 (0.65, 0.73) | 95/685 | 13.87 | 1.18 (0.87-1.59) | 1.18 (0.80-1.73) |
| Quartile3 (0.73, 0.80) | 83/690 | 12.03 | 0.92 (0.67-1.26) | 0.95 (0.62-1.46) |
| Quartile4 (0.80, 0.94) | 71/690 | 10.29 | 0.84 (0.61-1.16) | 0.83 (0.53-1.30) |
| *p* for Trend |  |  | 0.13 | 0.90 |
| Residential PM_2.5_ |  |  |  |  |
| Quartile1 (22.00, 28.70) | 11/689 | 1.60 | Ref. | Ref. |
| Quartile2 (28.70, 31.00) | 20/688 | 2.91 | 1.36 (0.65-2.84) | 1.47 (0.70-3.08) |
| Quartile3 (31.00, 33.80) | 37/688 | 5.38 | 1.65 (0.84-3.25) | 1.89 (0.96-3.72) |
| Quartile4 (33.80, 47.10) | 258/688 | 37.50 | 7.99 (4.34-14.72) | 10.62 (5.69-19.82) |
| *p* for Trend |  |  | <0.001 | <0.001 |

Abbreviations: CI, confidence interval; HR, hazard ratio; NDVI, normalized difference vegetation index; PM_2.5_, fine particulate matter.

^*^Age at the diagnosis date and sex were adjusted for in the Cox model.

^‡^Age at the diagnosis date, sex, occupation, county-level migrant population, drug resistance, residential nighttime light, distance to the nearest roads, and residential road density were adjusted for in the Cox model.**eTable2. Pulmonary tuberculosis and exposure to residential greenness and PM_2.5_, excluding follow-ups less than 90 days**

| Exposures | n/N | % | Adjusted  HR (95% CI) ^*^ | Adjusted  HR (95% CI) ^‡^ |
| --- | --- | --- | --- | --- |
|  |  |  |  |  |
| Residential NDVI |  |  |  |  |
| Quartile1 (0.39, 0.65) | 127/1599 | 7.94 | Ref. | Ref. |
| Quartile2 (0.65, 0.73) | 148/1577 | 9.38 | 1.07(0.84-1.35) | 1.06 (0.78-1.44) |
| Quartile3 (0.73, 0.79) | 140/1584 | 8.84 | 0.91 (0.72-1.16) | 0.89 (0.63-1.26) |
| Quartile4 (0.79, 0.94) | 114/1587 | 7.18 | 0.80 (0.62-1.02) | 0.76 (0.52-1.09) |
| *p* for Trend |  |  | 0.04 | 0.02 |
| Residential PM_2.5_ |  |  |  |  |
| Quartile1 (22.00, 29.00) | 18/1589 | 1.13 | Ref. | Ref. |
| Quartile2 (29.00, 31.40) | 37/1583 | 2.34 | 1.55 (0.88-2.73) | 1.74 (0.99-3.06) |
| Quartile3 (31.40, 33.90) | 62/1590 | 3.90 | 1.79 (1.06-3.03) | 2.13 (1.26-3.62) |
| Quartile4 (33.90 47.10) | 412/1584 | 26.01 | 9.20 (5.72-14.82) | 13.30 (8.19-21.62) |
| *p* for Trend |  |  | <0.001 | <0.001 |

Abbreviations: CI, confidence interval; HR, hazard ratio; NDVI, normalized difference vegetation index; PM_2.5_, fine particulate matter.

^*^Age at the diagnosis date and sex were adjusted for in the Cox model.

^‡^Age at the diagnosis date, sex, occupation, county-level migrant population, drug resistance, residential nighttime light, distance to the nearest roads, and residential road density were adjusted for in the Cox model.

**eTable3. The association of drug-resistant tuberculosis occurrence with exposure to residential greenness and PM_2.5_**

| Exposures | Adjusted OR (95% CI) ^*^ | Adjusted OR (95% CI) ^‡^ |
| --- | --- | --- |
| Residential NDVI |  |  |
| Quartile1 (0.39, 0.65) | Ref. | Ref. |
| Quartile2 (0.65, 0.73) | 1.61 (0.96-2.76) | 1.93 (0.99-3.83) |
| Quartile3 (0.73, 0.80) | 1.34 (0.78-2.34) | 1.69 (0.80-3.64) |
| Quartile4 (0.80, 0.94) | 1.20 (0.69-2.11) | 1.55 (0.70-3.48) |
| *p* for Trend | 0.76 | 0.82 |
| Residential PM_2.5_ |  |  |
| Quartile1 (22.00, 28.80) | Ref. | Ref. |
| Quartile2 (28.80, 31.20) | 1.73 (0.92-3.30) | 2.00 (1.07-3.85) |
| Quartile3 (31.20, 33.77) | 1.76 (0.96-3.35) | 2.22 (1.17-4.32) |
| Quartile4 (33.77, 47.10) | 3.24 (1.88-5.89) | 4.82 (2.58-9.38) |
| *p* for Trend | <0.001 | <0.001 |

Abbreviations: CI, confidence interval; NDVI, normalized difference vegetation index; OR, odds ratio; PM_2.5_, fine particulate matter.

^*^Age at the diagnosis date and sex were adjusted for in the logistic regression model.

^‡^Age at the diagnosis date, sex, occupation, county-level migrant population, residential nighttime light, distance to the nearest roads, and residential road density were adjusted for in the logistic regression model.**eTable4. The association of pulmonary tuberculosis recurrence with exposure to** **residential greenness and PM_2.5_**

| Exposures | n/N | % | Adjusted  HR (95% CI) ^*^ | Adjusted  HR (95% CI) ^‡^ |
| --- | --- | --- | --- | --- |
|  |  |  |  |  |
| Residential NDVI |  |  |  |  |
| Quartile1 (0.39, 0.65) | 127/1599 | 7.94 | Ref. | Ref. |
| Quartile2 (0.65, 0.73) | 148/1577 | 9.38 | 1.10 (0.87-1.38) | 1.14 (0.84-1.55) |
| Quartile3 (0.73, 0.79) | 140/1584 | 8.84 | 0.93 (0.73-1.18) | 0.88 (0.63-1.25) |
| Quartile4 (0.79, 0.94) | 114/1587 | 7.18 | 0.81 (0.64-1.04) | 0.66 (0.45-0.97) |
| *p* for Trend |  |  | 0.05 | 0.001 |
| Residential PM_2.5_ |  |  |  |  |
| Quartile1 (22.00, 29.00) | 18/1589 | 1.13 | Ref. | Ref. |
| Quartile2 (29.00, 31.40) | 37/1583 | 2.34 | 1.59 (0.88-2.88) | 2.14 (1.18-3.89) |
| Quartile3 (31.40, 33.90) | 62/1590 | 3.90 | 2.07 (1.20-3.59) | 3.38 (1.95-5.88) |
| Quartile4 (33.90 47.10) | 412/1584 | 26.01 | 10.52 (6.36-17.39) | 20.60 (12.31-34.45) |
| *p* for Trend |  |  | <0.001 | <0.001 |

Abbreviations: CI, confidence interval; HR, hazard ratio; NDVI, normalized difference vegetation index; PM_2.5_, fine particulate matter.

^*^Age at the diagnosis date and sex were adjusted for in the Cox model.

^‡^Age at the diagnosis date, sex, occupation, county-level migrant population, drug resistance, residential nighttime light, distance to the nearest roads, residential road density, Ozone, sulfur dioxide, temperature, and relative humidity were adjusted for in the Cox model.

**References**

1. Ge, E., et al., *Greenness exposure and all-cause mortality during multi-drug resistant tuberculosis treatment: A population-based cohort study.* Sci Total Environ, 2021. **771**: p. 145422.

2. Wei, J., et al., *Reconstructing 1-km-resolution high-quality PM2.5 data records from 2000 to 2018 in China: spatiotemporal variations and policy implications.* Remote Sensing of Environment, 2021. **252**: p. 112136.

3. Wei Jing, L.I.Z., *ChinaHighPM10: High-resolution and High-quality Ground-level PM10 Dataset for China (2000-2023)*, C. National Tibetan Plateau Data, Editor. 2024, National Tibetan Plateau Data Center.

4. Wei Jing, L.I.Z., *ChinaHighNO2: High-resolution and High-quality Ground-level NO2 Dataset for China (2008-2023)*, C. National Tibetan Plateau Data, Editor. 2024, National Tibetan Plateau Data Center.

5. Wei Jing, L.I.Z., *ChinaHighSO2: High-resolution and High-quality Ground-level SO2 Dataset for China (2013-2023)*, C. National Tibetan Plateau Data, Editor. 2024, National Tibetan Plateau Data Center.

6. Wei Jing, L.I.Z., *ChinaHighO3: High-resolution and High-quality Ground-level MDA8 O3 Dataset for China (2000-2023)*, C. National Tibetan Plateau Data, Editor. 2024, National Tibetan Plateau Data Center.

7. Shouzhang, P., *1 km multi-scenario and multi-model monthly temperature data for China (2021-2100)*, C. National Tibetan Plateau Data, Editor. 2025, National Tibetan Plateau Data Center.

8. Zhang, H., et al., *HiMIC-Monthly: A 1 km high-resolution atmospheric moisture index collection over China, 2003-2020.* Sci Data, 2024. **11**(1): p. 425.
